# Empowerment over enforcement: unpacking the psychological drivers of AI-assisted deep revision in EFL writing

**DOI:** 10.3389/fpsyg.2026.1871022

**Published:** 2026-09-10

**Authors:** Huan Li, Wenna Zhang

**Affiliations:** 1 School of Foreign Languages, Qiongtai Normal University, Haikou, China; 2 School of Educational Studies, Universiti Sains Malaysia, Penang, Malaysia; 3 School of Foreign Languages, Huaibei Normal University, Huaibei, China

**Keywords:** AI prompting literacy, cognitive load theory, deep revision engagement, EFL writing, external mandate, generative AI, self-determination theory

## Abstract

The rapid integration of generative artificial intelligence (AI) in higher education highlights a critical tension between top-down administrative enforcement and students’ genuine cognitive investment. Drawing upon Self-Determination Theory (SDT) and Cognitive Load Theory (CLT), this study investigates how AI Prompting Literacy (APL) and External Mandate (EM) differentially predict the Deep Revision Engagement (DRE) of English as a Foreign Language (EFL) learners. Utilizing survey data from 327 Chinese university students and partial least squares structural equation modeling (PLS-SEM), we tested a multiple parallel mediation model incorporating Perceived Competence (PC), Intrinsic Motivation (IM), and Psychological Safety (PS). The empirical results demonstrate that External Mandate yields a negligible direct path, suggesting that compliance-driven requirements may fail to directly stimulate deep cognitive participation. Conversely, AI Prompting Literacy emerges as a significant predictor within this structural model. Higher prompting literacy exhibits a positive indirect association with learners’ core psychological needs, thereby predicting an elevated level of Deep Revision Engagement through the parallel pathways of Perceived Competence, Intrinsic Motivation, and Psychological Safety. This research complements traditional technology acceptance frameworks by shifting the analytical lens toward internal psychological needs, suggesting that educational policies are more effective when focusing on cultivating student prompting capabilities alongside necessary institutional guidance.

## 1 Introduction

Artificial intelligence (AI) is reshaping higher education at an unprecedented speed, providing great potential for personalized learning and real-time feedback (Wei, 2023; Wessels, 2025). However, as generative artificial intelligence gradually shifts from experimental tools to institutional coercion, a key behavioral paradox has emerged: externally imposed use requirements may not automatically translate into students’ deep cognitive participation (Hopcan et al., 2024). In the field of English as a foreign language (EFL) writing, a vast majority of pioneering studies have heavily focused on technology acceptance paradigms, examining variables such as perceived ease of use, technical utility, and immediate writing outcomes (Almusharraf, 2026; Alwaqdani, 2025; Wei, 2023). While these studies offer valuable insights into initial tool adoption, they frequently treat AI interaction as a static, surface-level editing assistant, thereby leaving a distinct empirical gap regarding the sustained psychological mechanisms that drive learners’ “deep behavioral engagement,” specifically, the iterative, high-order cognitive processing required for autonomous text reconstruction (Crompton and Burke, 2023; Yuan et al., 2024). Consequently, it is worthwhile to empirically investigate what is associated with this deeper level of behavioral commitment beyond mere technical adoption.

Unlike mechanical language drills, academic writing is essentially an iterative process with high cognitive requirements, which requires continuous evaluation, synthesis, and improvement of ideas (Kellogg, 2008; Teng and Zhang, 2020). When AI tools are only introduced as compliance-driven tasks, this key critical reflection space is easily bypassed (Alwaqdani, 2025). Learners may use artificial intelligence superficially under the pressure of teachers, rather than spontaneously using it for text reconstruction (Crompton and Burke, 2023; Alwaqdani, 2025). This superficial obedience exposes a key theoretical blind spot in the current literature.

While the initial adoption rate and general usage frequency of educational technologies can be accurately predicted by the Technology Acceptance Model (TAM) (Liu et al., 2010), recent TAM-based empirical studies in the GenAI context primarily focus on variables such as “perceived ease of use” and “perceived usefulness” (e.g., Alwaqdani, 2025; Hopcan et al., 2024). Although these TAM-centric studies successfully explain whether learners will initially adopt an AI tool, they treat the technology largely as an external utility. Consequently, TAM is inherently limited in capturing the depth of cognitive investment during the actual learning process. Academic writing is not a one-off task but an iterative process of text reconstruction that demands sustained cognitive effort. TAM is inherently constrained in capturing the internal psychological nuances, such as the satisfaction of autonomy and competence, which are often considered vital for sustaining such high-level, cognitively demanding academic efforts. If students regard the integration of artificial intelligence merely as an easy shortcut or a top-down administrative burden, their autonomy may be compromised, which within alternative theoretical models, might explain variations in their qualitative depth of technical engagement. Therefore, a theoretical contribution of this study lies in supplementing the TAM paradigm by broadening the analytical lens from predicting initial, surface-level technology adoption toward investigating the psychological factors associated with deep behavioral engagement through the lens of Self-Determination Theory (SDT). Therefore, understanding how to stimulate students’ inner motivation instead of relying on External Mandate has become a critical pedagogical consideration in the AI era (Li and Thien, 2025).

While traditional technology-adoption paradigms, such as the Technology Acceptance Model (TAM) or the Unified Theory of Acceptance and Use of Technology (UTAUT), heavily prioritize perceived usefulness and administrative or social norms as core drivers of tool usage (Venkatesh et al., 2003), they frequently conceptualize technology adoption as a binary outcome (use or non-use). This conceptualization falls short when applied to complex, heuristic tasks like L2 academic text reconstruction, which involves intricate meta-cognitive loops of planning, evaluating, and executing rhetorical changes (Flower and Hayes, 1981). While traditional utilitarian software often operates on linear pathways, interaction with generative AI is frequently characterized by non-linear and iterative dynamics. Consequently, alternative frameworks rooted primarily in extrinsic utility may offer limited capacity to fully explain the deep qualitative variances in how students engage with automated feedback. By centering our inquiry on Self-Determination Theory (SDT), this study aims to move beyond surface-level adoption metrics, thereby addressing the potential psychological precursors that facilitate deep cognitive investment rather than mere technical compliance.

To address this gap and test the “empowerment over enforcement” dichotomy, this study draws upon Self-Determination Theory (SDT) to examine the sequential predictive associations between students’ interaction with generative AI. Specifically, this study aims to answer the following research questions:

*RQ1*: How does AI Prompting Literacy influence Deep Revision Engagement through the mediating roles of Perceived Competence, Intrinsic Motivation, and Psychological Safety?

*RQ2*: What is the direct impact of External Mandates on EFL learners’ Deep Revision Engagement?

## 2 Theoretical grounding and hypotheses

### 2.1 Self-determination theory

Self-Determination Theory (SDT) posits that human cognitive engagement and behavioral persistence are deeply contingent upon the satisfaction of three basic psychological needs: autonomy, competence, and relatedness (Ryan and Deci, 2020). Within educational technology paradigms, when these innate needs are supported by environmental affordances or personal resources, an individual’s motivational profile shifts from controlled regulation to autonomous regulation, thereby catalyzing deeper cognitive investment (Reeve, 2012; Jang et al., 2016).

However, within the specific domain of AI-assisted EFL writing, the phenomenological nature of these needs undergoes a distinct contextual shifting, necessitating a parsimonious scoping of the theoretical framework. Because the instructional setting investigated herein predominantly centers on a solitary, human-machine dialogic interaction rather than collaborative, human-to-human group dynamics, the traditional interpersonal need for “relatedness” becomes significantly less salient. Consequently, to maintain model parsimony and alignment with the empirical context, this study deliberately narrows its theoretical focus to the cognitive and motivational dimensions of SDT: Perceived Competence and Autonomous Motivation (operationally manifested as Intrinsic Motivation).

Furthermore, to capture how the psychological baseline for autonomous engagement is established during human-AI collaboration, this study introduces Psychological Safety as a critical environmental affordance that interacts with SDT mechanisms. Unlike human instructors or peers who may inadvertently evoke evaluative anxiety, generative AI provides a “zero-judgment,” low-stakes digital space. This uniquely supportive psychological climate buffers learners against the fear of negative evaluation, thereby securing the cognitive safety necessary for students to exercise genuine autonomy and risk-taking during text revision. Therefore, within this AI-assisted writing environment, SDT, complemented by psychological safety, provides a conceptual foundation for examining how student writing engagement is sustained through the dual pathways of cognitive mastery (Perceived Competence) and volitional drive (Intrinsic Motivation).

### 2.2 AI prompting literacy and psychological need satisfaction

AI Prompting Literacy (APL), conceptualized as a learner’s capacity to strategically formulate, evaluate, and iterate procedural instructions to optimize generative AI outputs, represents a critical personal resource in contemporary digital learning environments. According to Self-Determination Theory (SDT), the development and deployment of such personal competencies serve as vital precursors to psychological empowerment and structural motivation (Ryan and Deci, 2020). When students possess sophisticated prompting capabilities, they can exert precise agency over AI behavior, which is positively associated with their Perceived Competence (Su et al., 2023). Crucially, this strategic mastery transforms the human-AI interface from an unpredictable algorithmic black box into a highly controllable, responsive, and low-stakes dialogic space. By ensuring that algorithmic behaviors conform to user expectations, high prompting proficiency minimizes the evaluative anxiety typical of traditional peer-centered classrooms, thereby laying the psychological foundation for a sense of Psychological Safety.

To fully theoretize the mechanisms underlying this empowerment, Cognitive Load Theory (CLT; Sweller, 2011) provides a crucial, complementary framework that elucidates how APL substantively catalyzes the satisfaction of SDT’s psychological needs. In traditional second language (L2) writing tasks, learners frequently experience severe cognitive overload as they simultaneously navigate low-level linguistic mechanics (e.g., grammatical accuracy, lexical retrieval) and high-level rhetorical planning, a compounding pressure that suppresses their perceived efficacy. APL functions as a strategic cognitive scaffold within this dynamic. By expertly directing generative tools, learners can effectively offload repetitive, lower-level linguistic operations to the algorithm. This delegation potentially reduces extraneous cognitive load, defined as the mental effort wasted on task-extrinsic friction, thereby liberating vital working memory capacity, which represents the primary human cognitive system used for temporary information retention and processing (Sweller, 2011).

Crucially, within the integrated model proposed herein, this cognitive liberation (CLT) is sequentially linked to psychological need satisfaction (SDT). The newly freed-up working memory enables learners to reallocate their limited mental bandwidth toward high-level academic reflection, voice conceptualization, and structural text synthesis (Kellogg, 2008). This agentic shift, from struggling with technical language barriers to actively orchestrating algorithmic output, substantively supports the core psychological need for autonomy, thereby potentially fostering Intrinsic Motivation (IM). Furthermore, because sophisticated APL allows learners to establish a predictable and highly aligned human-computer feedback loop, it drastically reduces cognitive ambiguity and operational anxiety. This heightened predictability strengthens students’ cognitive agency and consistently consolidates their sense of Psychological Safety (PS) over the collaborative writing process. Accordingly, the following hypotheses are proposed:

*H1*: AI Prompting Literacy (APL) significantly impacts Perceived Competence (PC).

*H2*: AI Prompting Literacy (APL) significantly impacts Intrinsic Motivation (IM).

*H3*: AI Prompting Literacy (APL) significantly impacts Psychological Safety (PS).

### 2.3 Psychological mediators driving deep revision engagement

Deep Revision Engagement (DRE) refers to the iterative refinement of the logical structure, language style, and depth of content of a text by learners with the assistance of AI (Yuan et al., 2024). Unlike surface-level editing, such as unconditionally accepting automated grammar corrections, DRE requires a critical, dialogic interaction with the generative tool (Almusharraf, 2026). Under this collaborative framework, learners are expected to evaluate AI-generated alternatives, synthesize complex ideas, and ensure the final output authenticates their original authorial voice. This cognitively demanding behavior is more likely to be sustained when students feel competent (PC), derive intrinsic pleasure (IM), and are in a safe (PS) psychological environment (Reeve, 2012; Ryan and Deci, 2020). Because restructuring a draft demands sustained mental effort and a willingness to confront textual flaws, learners may benefit from the self-efficacy to judge the machine’s outputs (PC), the internal drive to pursue writing quality over mere task completion (IM), and the emotional security to view writing as an exploratory rather than an evaluative process (PS) (Kellogg, 2008). Lacking these psychological foundations, students may exhibit a higher probability of slipping into “algorithmic dependence,” potentially compromising their authorial agency to the AI for the sake of expediency (Selwyn, 2022).

*H4*: Perceived Competence (PC) significantly impacts Deep Revision Engagement (DRE).

*H5*: Intrinsic Motivation (IM) significantly impacts Deep Revision Engagement (DRE).

*H6*: Psychological Safety (PS) significantly impacts Deep Revision Engagement (DRE).

### 2.4 Limitations of external mandate

**Figure 1:**
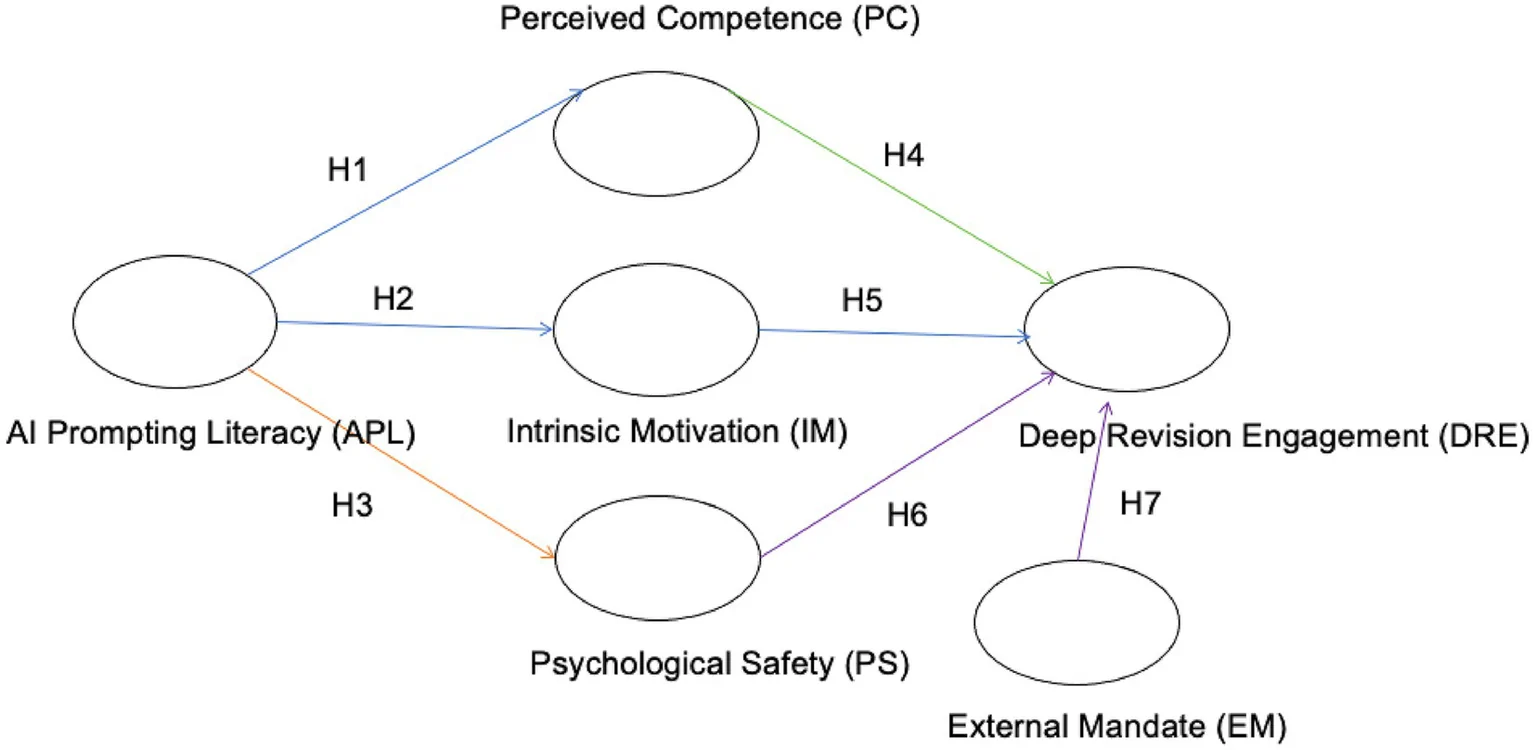
Proposes the research model.

Self-Determination Theory (SDT) defines External Mandate (EM) as a typical form of controlled motivation. Under this theoretical framework, such commands transform the learner’s perceived locus of causality from the inner desire to master the external pressure to meet the requirements of the system (Ryan and Deci, 2020). Historically, educational technology adoption paradigms relied heavily on external mandates, suggesting that external pressure could force student compliance (Deci and Ryan, 2000). However, in the contemporary era characterized by the immense popularity, accessibility, and powerful text-generation functions of AI, the explanatory power of these traditional “teacher enforcement” models is highly questionable. Given the strong spontaneous appeal of generative tools, it is necessary to empirically test whether pre-GenAI assumptions about top-down enforcement still hold true when it comes to high-level cognitive tasks. Therefore, this study sets out to examine whether traditional external mandates still possess any meaningful impact in an environment where students are already intrinsically drawn to the technology (Figure 1).

*H7*: External Mandate (EM) has a significant impact on Deep Revision Engagement (DRE).

## 3 Methods

### 3.1 Research design and sampling strategy

This study employed a quantitative research method based on a cross-sectional survey design, and selected the research subjects through purposive sampling. Since the aim of this study was to explore the deep adoption of artificial intelligence in English writing and its psychological mechanisms, the research subjects were mainly targeted at EFL learners in Chinese universities. This specific student cohort faces rigorous academic writing demands and possesses an established foundation in digital literacy, making them a highly suitable target population for investigating the integration of generative AI within English for Academic Purposes (EAP) writing contexts.

In order to enhance the representativeness of the sample and minimize the systematic bias caused by a single institutional background, this study recruited participants from two distinct types of higher education institutions in China: an application-oriented undergraduate institute of technology located in northern China and a regional normal university located in eastern China. These universities are particularly important to the focus of this study since they actively support the digital transformation of education and the reform of English instruction. According to the suggestion of Comrey and Lee (2013), the typical sample size for such empirical research is usually between 200 and 300.

To ensure linguistic equivalence, the original English scales underwent a rigorous translation and back-translation procedure (Brislin, 1970) conducted by two bilingual applied linguistics researchers. The recruitment of participants was executed on a strictly voluntary basis, designed to systematically eliminate potential social compliance bias or course-related pressure. To safeguard against subtle instructor-imposed coercion, regular course lecturers were excluded from the invitation and data collection process. The online survey link was distributed independently by the core research team through public institutional student notification forums following the conclusion of the academic semester. The recruitment materials explicitly stipulated that participation carried no monetary incentives or extra course credits, and participants were assured that electing to decline the survey would exert zero consequence on their course grading, evaluation, or academic standing. The survey was administered via an online questionnaire platform. To ensure data quality, explicit exclusion criteria were applied: responses that failed the embedded attention-check questions or were completed in less than 90 s were systematically excluded from the analysis. Given that the online survey system was configured to require responses for all items, the final dataset contained no missing data. Furthermore, a sample size validation was performed using G Power. To secure maximum conservative rigor, we parameterized a multiple linear regression framework targeting the most demanding segment of our structural model. Following Hair et al. (2019), the minimum sample size threshold for PLS-SEM estimation must be governed by the endogenous variable receiving the largest number of structural paths; in our proposed framework, the primary endogenous construct, Deep Revision Engagement (DRE), features exactly four incoming structural antecedents (PC, IM, PS, and EM). Conforming to this parameter specification, a multiple regression power analysis was configured with a medium effect size $f^{2}=0.15$ , an α error probability of 0.05, a statistical power of 0.95, and exactly four predictors. The power analysis indicated that a minimum sample size of 129 was statistically mandated to accurately detect stable relationships. Consequently, our final analytical dataset of 327 valid cases exceeds this empirical threshold, ensuring excellent statistical power for the subsequent structural model assessment.

During this data collection process, the research team retrieved a gross pool of 380 online questionnaires. A rigorous data screening and cleaning procedure was subsequently executed, resulting in the systemic attrition of exactly 53 invalid forms and yielding a final analytical sample of 327 valid responses (an effective recovery rate of 86.053%). The exact sample attrition flow across our three pre-established exclusion criteria was as follows: (1) 21 responses were eliminated due to attention-check failures, where participants missed the embedded verification instructions; (2) 18 responses were purged due to rapid completion times below the 90-s threshold, indicating automatic clicking without active reading; and (3) 14 responses were excluded for exhibiting clear response patterns. A response pattern was operationally defined as ‘straight-lining’ behavior, where a participant sequentially endorsed the exact same Likert-scale point (e.g., all 1 s or all 7 s) across more than 15 consecutive measurement items, thereby demonstrating an absolute absence of statistical variance and cognitive engagement. This final sample size (*N* = 327) far exceeded the minimum threshold calculated by G-Power, significantly enhancing the reliability and statistical power of the subsequent data analysis results. Among the 327 valid participants, the sample comprised 150 males (45.9%) and 177 females (54.1%). The participants’ ages ranged from 18 to 21 years, with a mean age of 19.62 years (SD = 0.84). In terms of academic background, the students were distributed across various disciplines, including Humanities and Social Sciences (*n* = 128, 39.1%), Science and Engineering (*n* = 141, 43.1%), and other majors (*n* = 58, 17.8%). At the time of the study, all participants were either freshmen or sophomores in a university actively enrolled in compulsory college English courses, with an average of 10.5 years of prior formal English learning experience.

### 3.2 Data collection procedure

All measurement tools used in this study are based on verified, mature scales and have been appropriately adapted to fit the specific context of generative AI-assisted second language writing. Specifically, AI Prompting Literacy (APL) was measured using a 3-item scale adapted from Su et al. (2023), designed to assess students’ proficiency in formulating and iterating prompts; a sample item includes: “I can accurately adjust my prompt instructions to guide the AI to output the desired text structure.” For the basic psychological needs under the SDT framework, three core sub-scales were adapted from classic motivation literature and relevant educational technology research (Chen et al., 2022; Deci and Ryan, 2000; May et al., 2004). Perceived Competence (PC) was operationalized as a 3-item construct, with the sample item: “I feel capable of critically evaluating and selecting the best feedback generated by AI.” Intrinsic Motivation (IM) comprised 4 items reflecting the psychological enjoyment of writing with AI, such as: “I find the process of using AI to explore different writing styles deeply interesting.” Psychological Safety (PS) was measured via a 5-item scale, with a sample item stating: “The non-judgmental environment of interacting with AI makes me feel safe to experiment with complex vocabulary.” Furthermore, Deep Revision Engagement (DRE) was measured using a 3-item scale adapted from Yuan et al. (2024), capturing high-level cognitive input and text reconstruction, exemplified by the item: “I actively synthesize AI suggestions with my own ideas to substantially restructure my writing drafts.”

The measurement of External Mandate (EM) was specifically developed for this study to reflect the actual teaching-management context in higher education, where instructors mandate the use of AI for academic purposes. To ensure content validity, the scale development procedure began with operationalizing the concepts of “mandated technology adoption” (Brown et al., 2002) and “external regulation” (Ryan and Deci, 2020) within the SDT framework. Following expert review and semantic refinement during the pilot study, the final EM scale was structured as a unidimensional construct comprising three specific items. The items are: (1) “My instructor explicitly requires me to use generative AI tools to complete my English writing assignments”; (2) “Using AI to generate or revise drafts is a mandatory part of the course evaluation/grading requirements”; and (3) “I use AI in my writing primarily because the teacher forces us to do so, rather than by my own spontaneous choice.” All items across the constructs were scored using a 7-point Likert scale, ranging from 1 (“strongly disagree”) to 7 (“strongly agree”).

As the first step of the process, the research team conducted pre-research (Pilot study) before officially distributing the questionnaires. In order to assess the scale’s initial reliability and semantic clarity, 105 EFL students from a selected university participated in this test. The core purpose of this pilot study was not only to test the initial reliability of the constructs but, more importantly, to refine, merge, or revise the measurement items based on participant feedback. Following the pilot test, specific adjustments were made to the questionnaire to enhance its content validity. First, two initially overlapping items within the ‘Psychological Safety’ construct were merged into a single item to eliminate redundancy and reduce survey fatigue. Second, the wording of one item in the newly developed ‘External Mandate’ scale was revised; pilot participants indicated that the distinction between ‘general teacher suggestions’ and ‘mandatory grading requirements’ was ambiguous, so the phrasing was adjusted to explicitly emphasize the ‘mandatory’ nature of the grading policy. The data of these 105 pilot participants were not included in the final main research analysis. After obtaining ethical approval from the relevant administrative departments of the two target universities, the formal survey questionnaire was distributed through the widely used online questionnaire platform “Wenjuanxing^1^” in China. The questionnaire’s first page stated clearly the academic purpose of the study and earnestly promised that all participant information would be kept completely private. In order to safeguard participant privacy as much as possible, the study team has implemented a rigorous data processing procedure.

To ensure measurement quality, this study evaluated both the historical validity of the mature scales and their empirical performance in the current context. In their original developmental studies, all adopted scales demonstrated excellent psychometric properties, with baseline internal consistencies typically exceeding the 0.80 threshold. After collecting the formal data, this study strictly evaluated the internal consistency of each measurement tool using Cronbach’s alpha coefficient. The results showed excellent reliability across all constructs: the alpha value of AI Prompting Literacy was 0.859, Perceived Competence was 0.799, Intrinsic Motivation was 0.960, Psychological Safety was 0.879, Deep Revision Engagement was 0.844, and External Mandate was 0.880. The Cronbach’s alpha values of all core latent variables were well above the acceptable critical value of 0.70. Furthermore, to provide sufficient evidence of structural validity, comprehensive metrics including Composite Reliability (CR), Average Variance Extracted (AVE) for convergent validity, and the Heterotrait-Monotrait (HTMT) ratio for discriminant validity, were systematically assessed and are detailed in section 4.1 (see Tables 1, 2).

**Table 1:** Assessment of the measurement model.

First-order constructs	APL	PC	IM	PS	DRE	EM	Cronbach’s alpha	Composite reliability(CR)	Average variance extracted (AVE)
AI prompting literacy (APL)							0.859	0.914	0.780
APL 1	0.868								
APL 2	0.918								
APL 3	0.863								
Perceived competence (PC)							0.799	0.897	0.707
PC 1		0.763							
PC 2		0.917							
PC 3		0.835							
Intrinsic motivation (IM)							0.960	0.980	0.891
IM 1			0.916						
IM 2			0.956						
IM 3			0.962						
IM 4			0.942						
Psychological safety (PS)							0.879	0.912	0.677
PS 1				0.716					
PS 2				0.859					
PS 3				0.898					
PS 4				0.759					
PS 5				0.867					
Deep revision engagement (DRE)							0.844	0.905	0.761
DRE 1					0.869				
DRE 2					0.907				
DRE 3					0.841				
External mandate (EM)							0.880	0.932	0.822
EM1						0.905			
EM2						0.961			
EM3						0.850			

APL, AI Prompting Literacy; PC, Perceived Competence; IM, Intrinsic Motivation; PS, Psychological Safety; DRE, Deep Revision Engagement; EM, External Mandate. The same applies below. All calculations were performed by the authors.

**Table 2:** Discriminant validity (HTMT0.90) for first-order construct.

Constructs	APL	PC	IM	PS	DRE	EM
APL						
PC	0.727					
IM	0.468	0.669				
PS	0.742	0.702	0.338			
DRE	0.657	0.655	0.681	0.518		
EM	0.057	0.168	0.072	0.084	0.088	

All calculations were performed by the authors.

### 3.3 Data analysis

The data analysis in this study was done using the SmartPLS 4 software, which was created especially for the Partial Least Squares Structural Equation Modeling (PLS-SEM). Following the methodological guidelines by Hair et al. (2019), PLS-SEM was purposefully preferred over Covariance-Based SEM (CB-SEM) for four distinct reasons. First, regarding the research objective, this study is predictive and exploratory, aiming to identify the key psychological drivers of deep revision engagement, whereas CB-SEM is strictly oriented toward theory confirmation. Second, in terms of *theoretical maturity*, although Self-Determination Theory is well-established, its specific application to the emergent context of generative AI prompting literacy represents a novel theoretical extension. PLS-SEM is highly recommended for such exploratory theory development. Third, regarding model characteristics, the proposed framework incorporates a complex parallel multiple-mediation structure (including competence, intrinsic motivation, and psychological safety). PLS-SEM is statistically superior in handling complex structural models without encountering the convergence issues typical of CB-SEM. Finally, concerning data conditions, PLS-SEM is a variance-based nonparametric method that does not require strict multivariate normal distributions, making it inherently more effective for the survey data collected in this study (Hair et al., 2019). The analysis of the data rigorously adhered to a two-phase evaluation process: the structural model was examined after the reflective measurement model was assessed (Hair et al., 2019).

The dimensions’ discriminant validity and convergent validity were primarily investigated during the measurement model evaluation stage. According to Hair et al. (2019), convergent validity was established when the following three conditions were satisfied: (1) the index reliability (factor loadings) was at least 0.70; (2) the composite reliability (CR) was at least 0.70; and (3) the average extracted variance (AVE) was at least 0.50. The discriminant validity was also evaluated using the heterotrait-monotrait ratio (HTMT). This ratio primarily analyzes the average correlations within the same term with the average correlations between items of distinct constructions. The establishment of discriminant validity may be confirmed when this ratio falls below the rigorous threshold of 0.90 (Henseler et al., 2015).

The study used the bootstrapping self-referential sampling technique to randomly select 10,000 sub-samples from the original dataset for the structural model testing stage that followed. The significance level of each structural path was ascertained by calculating the T values using this method (Hair et al., 2013). Path coefficients (β) explained variance (R^2^), and effect size (f^2^) were among the primary quantitative indicators that were thoroughly investigated during the analysis (Thien and Jamil, 2020). Preacher and Hayes (2008) recommended using the Bootstrap self-referential sampling technique to rigorously assess the effects in the mediating models because the model contained multiple mediating variables (competence, intrinsic motivation, and psychological security).

## 4 Results

Prior to evaluating the structural model, structural collinearity and discriminant validity were assessed. Following the standard guidelines of Bagozzi et al. (1991), the correlation matrix was examined; all HTMT ratios fell safely below the 0.90 threshold, confirming discriminant validity. Furthermore, structural collinearity diagnostics indicated that all inner Variance Inflation Factor (VIF) values ranged from 1.000 to 2.230, comfortably below the recommended threshold of 5.0 (Hair et al., 2019), demonstrating the absence of structural multicollinearity. However, because this investigation relies strictly on a cross-sectional, self-report design within a single time point, these diagnostic metrics cannot entirely eliminate the potential threat of Common Method Bias (CMB). Consequently, CMB must be explicitly treated as an inherent methodological limitation of this research design, and all structural pathways, particularly the parallel mediation trajectories, are interpreted as model-dependent indirect associations rather than causal or chronological mediation links. To assess the overall model fit, the Standardized Root Mean Square Residual (SRMR) was calculated at 0.096, which satisfies the acceptable criterion of < 0.10 for PLS-SEM (Benitez et al., 2020).

### 4.1 Measurement model assessment

The evaluation of the measurement model for the first-order construct is shown in Table 1. Every construct’s convergent validity and internal consistency were confirmed. Each latent variable’s composite reliability (CR) was above 0.7, and the average extracted variance (AVE) exceeded the suggested cutoff point of 0.50 (Hair et al., 2013).

The discriminant validity (HTMT0.90) for each construct is shown in Table 2. The model has good discriminant validity since, as Table 2 illustrates, the heterotrait-monotrait ratio (HTMT) between all latent variables was the greatest at just 0.742, well below the strict threshold limit of 0.90 (Henseler et al., 2015).

### 4.2 Assessment of structural model and hypothesis testing

The test results of the structural model revealed significant psychological driving mechanisms. The model successfully explained 50.0% of the variance in Deep Revision Engagement (*R*^2^ = 0.500).

AI Prompting Literacy (APL) and Perceived Competence (PC) were found to be significantly positively correlated, supporting hypothesis H1 (*β* = 0.636, *t* = 24.387, *p* < 0.001). H1 was supported since zero was not included in the 95% confidence interval (CI) with bias adjustment. AI Prompting Literacy (APL) also showed substantial positive associations with Intrinsic Motivation (IM) (*β* = 0.439, *t* = 7.050, *p* < 0.001) and Psychological Safety (PS) (*β* = 0.657, *t* = 17.093, *p* < 0.001), supporting H2 and H3, respectively (Table 3).

**Table 3:** Results of structural model evaluation and hypothesis testing.

Hypothesis	Beta	Standard deviation (SD)	*t*-value	*p*-values	95% CI	Effect size	Decision
H1: APL - > PC	0.636	0.026	24.387	<0.001	[0.589, 0.693]	0.680	Supported
H2: APL - > IM	0.439	0.062	7.050	<0.001	[0.317, 0.561]	0.239	Supported
H3: APL - > PS	0.657	0.038	17.093	<0.001	[0.579,0.729]	0.761	Supported
H4: PC - > DRE	0.218	0.073	2.964	0.003	[0.069,0.356]	0.042	Supported
H5: IM - > DRE	0.448	0.066	6.765	<0.001	[0.321,0.580]	0.251	Supported
H6: PS - > DRE	0.181	0.069	2.633	0.008	[0.046,0.316]	0.042	Supported
H7: EM - > DRE	−0.013	0.060	0.165	0.869	[−0.126,0.109]	0.000	Not supported
APL - > PC - > DRE	0.139	0.046	2.983	0.003	[0.041,0.223]		Supported
APL - > IM - > DRE	0.197	0.047	4.166	<0.001	[0.117,0.301]		Supported
APL - > PS - > DRE	0.119	0.045	2.625	0.009	[0.031,0.212]		Supported

CI, Confidence Interval; APL, AI Prompting Literacy; PC, Perceived Competence; IM, Intrinsic Motivation; PS, Psychological Safety; EM, External Mandate; DRE, Deep Revision Engagement. All calculations were performed using SmartPLS 4.0. All calculations were performed by the authors.

The investigation indicated substantial positive correlations between the psychological variables and Deep Revision Engagement (DRE). Perceived Competence was a significant positive predictor of Deep Revision Engagement (*β* = 0.218, *t* = 2.964, *p* = 0.003), hence corroborating H4. Both Intrinsic Motivation (*β* = 0.448, *t* = 6.765, *p* < 0.001) and Psychological Safety (*β* = 0.181, *t* = 2.633, *p* = 0.008) positively predict Deep Revision Engagement, as their respective corrected bias 95% CI did not include zero, thus corroborating H5 and H6.

In contrast, no significant relationship was found between External Mandate (EM) and Deep Revision Engagement (*β* = −0.013, *t* = 0.165, *p* = 0.869), since the 95% CI encompasses zero, hence not supporting H7.

The effect size (f^2^) quantifies the impact within the structural model. Cohen (2013) classifies the effect sizes as small, moderate, and high, with cut-off values of 0.02, 0.15, and 0.35, respectively. This research suggests that AI Prompting Literacy significantly predicts Psychological Safety (*f*^2^ = 0.761) and Perceived Competence (*f*^2^ = 0.680), while showing a moderate predictive effect on Intrinsic Motivation (*f*^2^ = 0.239). Intrinsic Motivation moderately predicts Deep Revision Engagement (*f*^2^ = 0.251). The effects of Perceived Competence (*f*^2^ = 0.042) and Psychological Safety (*f*^2^ = 0.042) on Deep Revision Engagement are categorized as weak. Conversely, the effect size for the direct path of External Mandate is negligible (*f*^2^ = 0.000).

To evaluate the mediation claims, we assessed the specific indirect effects using a 10,000-resample bootstrapping procedure. The results demonstrated significant mediating roles for all three psychological variables. Specifically, the indirect path from AI Prompting Literacy (APL) to Deep Revision Engagement (DRE) through Perceived Competence (PC) was significant (*β* = 0.139, *t* = 2.983, *p* = 0.003). Similarly, the specific indirect effects through Intrinsic Motivation (IM) (*β* = 0.197, *t* = 4.166, *p* < 0.001) and Psychological Safety (PS) (*β* = 0.119, *t* = 2.625, *p* = 0.009) were also positive and statistically significant. These findings confirm the complex multiple-mediation mechanisms within the structural model.

**Figure 2:**
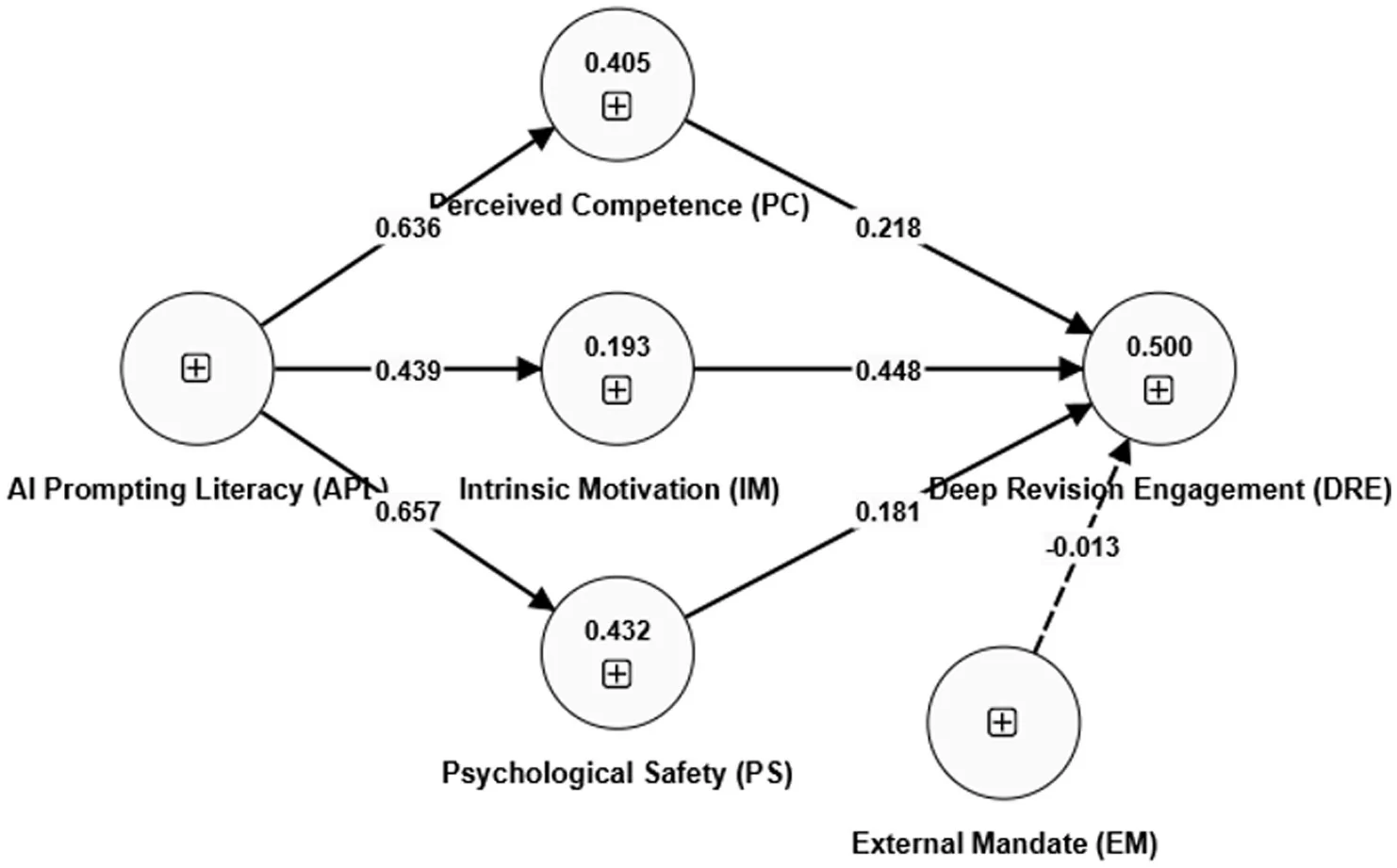
The structural model.

The structural model is shown in Figure 2.

## 5 Discussion

The findings of this study offer significant behavioral science insights for the contemporary implementation of artificial intelligence technologies in higher education. This study clarifies the detailed psychological mechanisms that are statistically associated with how learners interact with generative tools, rather than treating technology acceptance as a simple binary outcome.

First, the data show that AI Prompting Literacy (APL) is a significant positive predictor of Perceived Competence, Intrinsic Motivation, and Psychological Safety (H1, H2, H3). This explicitly moves beyond the previous conceptualization of technology as a mere “tool” (Hopcan et al., 2024). Instead, APL exhibits a strong positive association with student cognitive agency within this model specification. By learning to formulate, iterate, and refine prompts, students transition from passive recipients of algorithmic outputs to active co-creators of knowledge (Bozkurt et al., 2023). In the digital age, APL has emerged as the core psychological empowerment resource for EFL learners. When students master how to dialogue efficiently with AI, they regain control of the writing process, which is the driving force behind all deep learning behaviors (Su et al., 2023). Crucially, this mastery alleviates the extraneous cognitive overload typically associated with L2 academic writing, thereby freeing working memory capacity for higher-order tasks such as rhetorical reconstruction and critical synthesis (Almusharraf, 2026).

Second, the structural model suggests that Perceived Competence, Intrinsic Motivation, and Psychological Safety are key mediating factors that positively predict Deep Revision Engagement (H4, H5, H6). In traditional L2 writing, students often experience severe evaluation anxiety (Li and Thien, 2025) and linguistic paralysis. However, generative AI provides a “zero-judgment” dialogic space (Chen et al., 2022). Unlike human peer reviewers or instructors, whose feedback may unintentionally evoke face-threatening concerns, the conversational agent offers continuous, iterative feedback without social repercussions (Fryer et al., 2019). The elimination of this psychological burden (Psychological Safety), coupled with the heightened sense of capability derived from technological empowerment (Perceived Competence), allows students to experience genuine creative pleasure (Intrinsic Motivation). This psychological synergy potentially supports the cognitively demanding behavior of deep revision. This dynamic aligns with Krashen's (1982) Affective Filter Hypothesis, contextualized for the AI era: lowered emotional barriers exhibit a positive association with cognitive flexibility indicators within our model, which is hypothesized to support learners when experimenting with complex syntax and structural synthesis without the paralyzing fear of failure (Chiu, 2023).

Finally, the non-significant direct path from External Mandate (EM) to Deep Revision Engagement (H7) warrants a nuanced, dialectical evaluation. Conforming to a balanced administrative perspective, it is plausible to consider that top-down institutional mandates and compliance monitoring serve legitimate and foundational purposes in contemporary higher education. Administrative requirements are commonly enacted to secure educational equity by ensuring that all students, regardless of initial socioeconomic or technological backgrounds, are structurally introduced to emerging digital tools; furthermore, they can play an important role in preserving assessment validity, ensuring curriculum alignment, and maintaining instructional transparency. Therefore, our empirical findings do not suggest that institutional enforcement represents an administrative failure. Rather, the non-significant trajectory suggests that compliance-oriented policies may be insufficient on their own to stimulate high-level cognitive investment unless proactively integrated with student agency, specialized AI prompting literacy, and formative pedagogical scaffolding. Within the architectural lens of Self-Determination Theory (SDT), mandatory requirements are theorized to operate as controlled regulation, which appears capable of sustaining baseline operational compliance. However, to better facilitate the transition of this baseline usage into deep behavioral text reconstruction, top-down enforcement is predicted to be more effective when paired with capacity-building resources that support students’ internal locus of causality, enabling them to transition from passive compliance to autonomous epistemic engagement.

Crucially, this empirical reality could be interpreted through the lens of contemporary academic integrity and ethical concerns within generative AI scholarship. Critics frequently warn that the unchecked proliferation of generative tools in higher education threatens student authorship, potentially encouraging plagiarism, unauthorized synthesis, and intellectual passivity (Macfarlane, 2024; Sullivan et al., 2023). However, our non-significant path from External Mandate to Deep Revision Engagement (H7) offers a powerful counter-narrative to these pessimistic assumptions. When institutions attempt to mitigate ethical risks by enforcing rigid, monitoring-based AI compliance or transactional usage metrics, it may yield limited efficacy in triggering meaningful learning. In fact, top-down enforcement in the absence of specialized competence may backfire, potentially driving students toward ‘algorithmic dependence’ where they might surrender their authorial agency to secure a grade. Therefore, our correlational findings suggest that addressing the AI ethics crisis may require balancing necessary administrative frameworks with proactive pedagogical support, fostering AI prompting literacy (APL) so that students view the technology as a formative interlocutor rather than an ethical shortcut.

When AI usage is forced from the top down, students perceive the intervention as an administrative hurdle rather than a cognitive opportunity. As classic Self-Determination Theory (SDT) experimental evidence suggests, although controlled motivation can sustain engagement in simple, algorithmic tasks, it severely undermines performance in complex, heuristic endeavors like academic text reconstruction (Deci and Ryan, 2000). In the context of generative tools, this deprivation of autonomy manifests directly as “algorithmic dependence.” Under administrative pressure, learners prioritize efficiency over quality, deliberately bypassing the intellectually taxing process of critical dialogue. Instead of actively examining, refuting, or synthesizing the machine’s output, they surrender their authorial agency. Therefore, educational stakeholders are encouraged to critically reconsider monitoring-based, mandatory AI requirements. Instead, policies should prioritize the cultivation of students’ prompting literacy and the creation of a psychologically safe, autonomy-supportive environment to genuinely foster deep cognitive engagement.

This empirical result is consistent with core SDT tenets: externally controlled motivation may constrain students’ autonomy, thereby potentially limiting the opportunity for deep learning (Ryan and Deci, 2020). Compulsory requirements can, at best, elicit surface-level compliance; they may yield limited efficacy in fostering the intrinsic motivation required to critically optimize the deep structure of a text. When students perceive AI integration as an administrative obstacle rather than a self-endorsed learning strategy, they tend to adopt a ‘satisficing’ approach, performing only the minimum operations necessary to satisfy scoring rubrics.

## 6 Conclusion and implications

This study extends the discussion of artificial intelligence acceptance to a deeper level of psychological motivation. By integrating Self-Determination Theory (SDT) into the context of generative AI tools, this study complements existing literature by extending the analytical focus from simple technical utility toward the internal psychological experiences of learners. The empirical results suggest that “compulsory requirements” may yield limited efficacy in AI-assisted EFL writing. When institutions treat AI adoption merely as an administrative checklist, it inadvertently deprives learners of autonomy, resulting in superficial compliance rather than genuine skill acquisition. A significant statistical predictor for deep cognitive engagement stems from enhancing students’ AI Prompting Literacy (APL) and satisfying their basic psychological needs.

Specifically, when students master sophisticated prompting skills, they transition from passive consumers of machine-generated text to active directors orchestrating their own revision process. University administrators and educators are encouraged to carefully re-evaluate monitoring-centric technology policies obsessed with usage metrics or AI-detection software. Instead, the focus could pivot to AI prompting workshops and the construction of highly fault-tolerant academic evaluation systems. Crucially, this means formative feedback could take precedence over summative assessment. Curricula could be redesigned to explicitly teach human-AI collaboration as a core academic competency, evaluating the quality of the iterative dialogue between students and AI rather than just the final written product.

To better align these macro-theoretical insights with instructional practices, our empirical model suggests that EFL writing instruction may benefit from balancing broader technology configurations with adaptive, student-centered micro-scaffolding. Specifically, we propose a “Collaborative Human-AI Revision Design” that systematically embeds prompt engineering into the drafting, feedback, and rewriting phases. During the drafting stage, prompting should be explicitly taught as a cognitive support strategy, not a shortcut for text generation. Teachers should guide learners to construct “constraint-based prompts,” for instance, instructing the AI to generate structural outlines, thematic vocabulary matrices, or counter-arguments, while strictly prohibiting the generation of continuous prose.

In the subsequent feedback stage, the pedagogical goal shifts to fostering critical and conversational interaction. Students should be trained to use “criteria-specific diagnostic prompts” rather than passively capitulating to automated grammar corrections. By positioning the AI as a formative peer reviewer rather than an authoritative oracle, this process establishes a psychological safety zone. Learners can repeatedly experiment with complex syntactic structures and rhetorical strategies, receiving instant feedback on high-level academic concerns without the fear of negative human evaluation.

In the rewriting stage, the course could mandate critical synthesis. The evaluation focus could primarily center on how students negotiate with the AI’s feedback. Traditional summative scoring rubrics that heavily penalize linguistic inaccuracy exhibit a potential association with students slipping into “algorithmic dependence” in pursuit of higher grades. To dismantle this performance-oriented compliance, assessments could become process-oriented, for example, through “Prompt Reflection Portfolios.” Teachers should evaluate the metacognitive quality of the students’ interaction history: How did they iteratively refine their prompts? Which AI suggestions were critically rejected, and why? By actively rewarding “productive failure,” instances where students attempt complex prompts, critically analyze suboptimal AI outputs, and subsequently adjust their linguistic strategies, educators can structurally ensure that intrinsic motivation and critical thinking remain at the core of the AI-assisted writing classroom. Pedagogical empowerment, rather than administrative enforcement, appears to offer a more promising pathway toward supporting the academic potential of EFL learners in the GenAI era, ensuring that technology amplifies human intellect rather than bypassing it.

## 7 Limitations and future research

This research has specific limitations. The study is based on cross-sectional self-reporting data, because all latent constructs were measured simultaneously via single-source student questionnaires, the data are susceptible to potential Common Method Bias (CMB). While discriminant validity and inner VIF diagnostics fell within standard acceptable boundaries, future research should implement multi-wave longitudinal designs, mixed-method approaches, or paired teacher-student dyadic data to more isolate these indirect associations from method-induced variance. Secondly, while this study presents the demographic breakdown of the sample, it does not empirically model the moderating paths or multi-group variances of demographic characteristics (such as gender differences) within the structural model. Future studies may incorporate individual personality traits into the model to improve the cross-cultural applicability of the research results. Third, since deep revision participation is a highly complex and iterative cognitive process, relying on quantitative scales alone may not be able to fully capture the dynamic nuances of human-computer interaction. Therefore, future research can encourage the adoption of qualitative and behavioral tracking methods, such as “thinking” protocols or digital log data analysis. These methodological extensions will enable researchers to cross-verify the learners’ self-reported psychological state and their practical behavior.

## Data Availability

The original contributions presented in the study are included in the article/Supplementary material, further inquiries can be directed to the corresponding author.

## Footnotes

1
www.wjx.cn
