# Supplementary material for: Empowerment over enforcement: unpacking the psychological drivers of AI-assisted deep revision in EFL writing
Source: Front Psychol. 2026 Sep 10;17:1871022. doi: 10.3389/fpsyg.2026.1871022 (PMC13601272; doi:10.3389/fpsyg.2026.1871022)
Supplement: Supplementary file 1 [file Data_Sheet_1.PDF]

Appendix A: Measurement Scales

Note: All items were measured on a 7-point Likert scale ranging from 1 ("Strongly Disagree") to 7 ("Strongly Agree"), adapted from previous validated scales and contextualized for AI-assisted EFL writing.

AI Prompting Literacy (APL)

APL 1: I know how to formulate precise and constraint-based prompts to guide generative AI in modifying my English writing.

APL 2: I can accurately adjust my prompt instructions to guide the AI to output the desired text structure.

APL 3: I possess the necessary skills to instruct AI to focus on specific academic aspects of my writing (e.g., logical structure, thematic vocabulary, coherence).

Perceived Competence (PC)

PC 1: I feel capable of critically evaluating and selecting the best feedback generated by AI.

PC 2: Utilizing AI as a cognitive scaffold makes me feel more competent in tackling complex L2 academic writing tasks.

PC 3: I am confident in my epistemic ability to decide which AI-generated revisions to accept, modify, or completely reject.

Intrinsic Motivation (IM)

IM 1: I find the process of deeply revising my writing through human-AI interaction to be highly enjoyable.

IM 2: I find the process of using AI to explore different writing styles deeply interesting.

IM 3: Dialoguing with AI to continuously refine my text is an inherently rewarding learning experience for me.

IM 4: I feel a deep sense of personal satisfaction when I successfully collaborate with AI to enhance the intellectual depth of my draft.

Psychological Safety (PS)

PS 1: The non-judgmental environment of interacting with AI makes me feel safe to experiment with complex vocabulary.

PS 2: I do not experience anxiety or fear of negative evaluation when using AI to test out preliminary or imperfect writing ideas.

PS 3: The AI environment allows me to take rhetorical risks in my writing without the fear of embarrassment or peer pressure.

PS 4: I feel psychologically comfortable expressing my initial thoughts to the AI, knowing the interaction is private and non-threatening.

PS 5: Interacting with AI eliminates the emotional burden typically associated with traditional human grading or peer review.

Deep Revision Engagement (DRE)

DRE 1: I consistently engage in multiple rounds of iterative dialogue with AI to fundamentally restructure the logic and flow of my essays.

DRE 2: Rather than passively accepting automated grammar corrections, I utilize AI to critically synthesize and elevate complex ideas in my text.

DRE 3: I actively synthesize AI suggestions with my own ideas to substantially restructure my writing drafts.

External Mandate (EM)

EM 1: My instructor explicitly requires me to use generative AI tools to complete my English writing assignments

EM 2: Using AI to generate or revise drafts is a mandatory part of the course evaluation/grading requirements

EM 3: I use AI in my writing primarily because the teacher forces us to do so, rather than by my own spontaneous choice.
