# Supplementary material for: Empowerment over enforcement: unpacking the psychological drivers of AI-assisted deep revision in EFL writing
Source: Front Psychol. 2026 Sep 10;17:1871022. doi: 10.3389/fpsyg.2026.1871022 (PMC13601272; doi:10.3389/fpsyg.2026.1871022)
Supplement: Supplementary file 2 [file Table_1.DOCX]

Supplementary Material

# Abbreviations

| AI | Artificial Intelligence |
| --- | --- |
| APL | AI Prompting Literacy |
| AVE | Average Variance Extracted |
| CI | Confidence Interval |
| CMB | Common Method Bias |
| CR | Composite Reliability |
| DRE | Deep Revision Engagement |
| EFL | English as a Foreign Language |
| EM | External Mandate |
| HTMT | Heterotrait-Monotrait Ratio |
| IM | Intrinsic Motivation |
| L2 | Second Language |
| PC | Perceived Competence |
| PLS-SEM | Partial Least Squares Structural Equation Modeling |
| PS | Psychological Safety |
| SD | Standard Deviation |
| SDT | Self-Determination Theory |
| TAM | Technology Acceptance Model |
| VIF | Variance Inflation Factor |

The following abbreviations are used in this manuscript:

# Appendix A. Measurement Scales

Note: All items were measured on a 7-point Likert scale ranging from 1 ("Strongly Disagree") to 7 ("Strongly Agree"), adapted from previous validated scales and contextualized for AI-assisted EFL writing.

**Table A1.** Construct measurement items.

| **Construct & Source** | **Code** | **Measurement Items** |
| --- | --- | --- |
| AI Prompting  Literacy (APL) | APL 1 | I know how to formulate precise and constraint-based prompts to guide generative AI in modifying my English writing. |
|  | APL 2  APL3 | I can effectively adjust and iterate my prompts based on the initial feedback provided by the AI.  I possess the necessary skills to instruct AI to focus on specific academic aspects of my writing (e.g., logical structure, thematic vocabulary, coherence) |
| Perceived Competence (PC) | PC 1 | I feel highly capable of critically evaluating and filtering the writing suggestions generated by AI. |
|  | PC 2 | Utilizing AI as a cognitive scaffold makes me feel more competent in tackling complex L2 academic writing tasks. |
|  | PC 3 | I am confident in my epistemic ability to decide which AI-generated revisions to accept, modify, or completely reject. |
| Intrinsic Motivation (IM) | IM 1 | I find the process of deeply revising my writing through human-AI interaction to be highly enjoyable. |
|  | IM 2 | I engage in AI-assisted revision because I am genuinely interested in elevating the quality of my writing, not merely to complete an assignment. |
|  | IM 3 | Dialoguing with AI to continuously refine my text is an inherently rewarding learning experience for me. |
|  | IM 4 | I feel a deep sense of personal satisfaction when I successfully collaborate with AI to enhance the intellectual depth of my draft. |
| Psychological Safety (PS) | PS 1 | I feel completely safe making structural or linguistic mistakes when interacting with AI because it provides a "zero-judgment" space. |
|  | PS 2 | I do not experience anxiety or fear of negative evaluation when using AI to test out preliminary or imperfect writing ideas. |
|  | PS 3 | The AI environment allows me to take rhetorical risks in my writing without the fear of embarrassment or peer pressure. |
|  | PS 4 | I feel psychologically comfortable expressing my initial thoughts to the AI, knowing the interaction is private and non-threatening. |
|  | PS 5 | Interacting with AI eliminates the emotional burden typically associated with traditional human grading or peer review. |
| Deep Revision Engagement (DRE) | DRE 1 | I consistently engage in multiple rounds of iterative dialogue with AI to fundamentally restructure the logic and flow of my essays. |
|  | DRE 2 | Rather than passively accepting automated grammar corrections, I utilize AI to critically synthesize and elevate complex ideas in my text. |
|  | DRE 3 | I actively negotiate with AI-generated alternatives to ensure the final output authentically preserves my original authorial voice. |
| External Mandate (EM) | EM 1 | I use generative AI in my academic writing primarily because it is a top-down, mandatory requirement imposed by my instructor or institution. |
|  | EM 2 | My decision to interact with AI tools is heavily driven by the administrative pressure to meet minimum course requirements or avoid grade penalties. |
|  | EM 3 | I perceive the integration of AI in this writing course more as a compulsory administrative compliance rather than a self-endorsed learning strategy. |
